# The effectiveness of a WeChat-based multimodal nursing program for women with breast cancer: A randomized controlled trial protocol

**DOI:** 10.1097/MD.0000000000023526

**Published:** 2020-12-24

**Authors:** Na Zhao, Fang Yin, Xiaofang Wu, Yuxia Zhong

**Affiliations:** Department of Operating Room, The Second Affiliated Hospital of Hainan Medical University, Hainan, 570311, China.

**Keywords:** breast cancer, multimodal nursing program, protocol, WeChat

## Abstract

**Background:**

Breast cancer is one of the most familiar malignant tumor all over the world in women. The main treatment for the early breast cancer is surgery accompanied by the adjuvant therapy. Nevertheless, these treatments can lead to adverse reactions including sleep disturbances, fatigue, and pain. For our research, the objective is to assess the therapeutic effect of nursing program based on WeChat for the female breast cancer.

**Methods:**

This is a randomized controlled research, and it will be carried out from November 2020 to May 2021, which was granted via the Ethics Committee of the Second Affiliated Hospital of Hainan Medical University (LW2020056). In this study, patients meeting the following criteria will be included:

(1) patients aged 18 or older,
(2) stage I to III disease,
(3) patients newly diagnosed with the breast cancer, and
(4) patients undergoing surgery accompanied by adjuvant therapy.

And patients with

(1) mental or cognitive disorders and
(2) other malignancies will be excluded.

In intervention group, the patients are given multi-mode nursing program based on WeChat and routine nursing. In control group, patients are given routine nursing, involving vital signs monitoring, the education of health, the monitoring of postoperative complications, as well as postoperative drainage tube nursing. The main outcomes are the change of Functional Assessment of Cancer Therapy-Breast version 4.0 (FACT-Bv4.0) score. While the secondary result is the numerical rating scale (NRS).

**Results:**

Table 1 shows the clinical results between study group and control group.

**Conclusion:**

This paper has guided nurses to develop an evidence-based protocol to improve patient care for postoperative women with breast cancer.

**Trial registration:**

This study protocol was registered in Research Registry (researchregistry6180).

## 1 Introduction

Over the past several years, the increase of incidence rate of cancer and its influence on distinct psychological, physiological, and social levels in human life make it a vital issue in this century.^[1]^ In developed countries, incidence of breast cancer ranges from 1% to 2%, with approximately 5% yearly increase in less developed countries.^[2]^ It is estimated that more than 7 million people worldwide die from the cancer. The number of novel cancer cases is projected to rise from 15 million to 20 million by 2025.^[3]^ Furthermore, breast cancer is the most familiar kind of malignant tumor in women, with more than 1 million new cases annually.^[4,5]^

Breast cancer is a kind of tissue cancer, and chiefly involving the ducts, and the inner layer of lobules or milk glands.^[6]^ The main risk factors for the cancer involve race, high levels of hormone, age, dietary iodine deficiency as well as economic status.^[7,8]^ The breast cancer affects approximately 1/8 of the female population. Most of the time, thorough tissue removal, radiotherapy, chemotherapy, and the hormone therapy are necessary.^[9–12]^ Nevertheless, these treatments can lead to adverse reactions including sleep disturbances, fatigue, and pain. Such effects can significantly worsen the life quality of patients, especially in the periods after receiving surgery accompanied by the adjuvant treatment. Some methods have been utilized for the improvement of the life quality for female breast cancer patients. Previous studies have indicated that WeChat appears to be effective in improving postoperative nursing.^[13,14]^ However, few studies reported the use of WeChat in breast cancer. Therefore, we perform this protocol to assess the therapeutic effect of nursing program based on WeChat for the female breast cancer.

## 2 Methods

### 2.1 Study design

It is a randomized controlled experiment to be conducted from November 2020 to May 2021. It was permitted through the Ethics Committee of the Second Affiliated Hospital of Hainan Medical University (LW2020056), and this experiment was registered with research registry (researchregistry6180).

### 2.2 Inclusion and exclusion criteria

100 patients are included in the trial. In this study, patients meeting the following criteria will be included:

(1) patients aged 18 or older,
(2) stage I to III disease,
(3) patients newly diagnosed with the breast cancer, and
(4) patients undergoing surgery accompanied by adjuvant therapy.

And patients with

(1) mental or cognitive disorders and
(2) other malignancies will be excluded.

Sequence of random numbers is generated by a computer. Sequentially numbered sealed opaque envelopes are used for the concealment of random numbers. All patients participating in our experiment are randomly assigned to the study group and the control group, each group with 50 members.

### 2.3 Intervention

In intervention group, the patients are given multi-mode nursing program based on WeChat and routine nursing, the details are as follows:

(1) Physical rehabilitation:
  a. Offer the personalized information: adverse reactions of the adjuvant therapy; postoperative complications; complications of an implantable venous access or the peripheral insertion of central catheter.
  b. Self-monitoring and prevention of recurrence after discharge.
  c. Drawing up and then updating the patient-oriented activity, rest, and diet plans.
  d. Dealing with the fatigue and lack of sleep: engaging in physical activity and relaxation exercise (such as meditation, listening to music, muscle relaxation).
(2) Psychological rehabilitation:
  a. Conducting demand-oriented psychological counseling.
  b. Family (face-to-face guidance by professionals): meeting the patients’ needs; understanding their feelings; and accompanying them as much as possible.
  c. Peers (face to face guidance by professionals): sharing relevant experiences in coping with the negative mental state.
(3) Social rehabilitation: Role transformation: Gradually accomplishing the transformation of role from the patient to social role; and fulfill their original social role.

The project team based on WeChat involves 3 researchers and 1 postgraduate student majoring in breast cancer nursing, and 1 nurse and 1 doctor from the surgical department of breast cancer. All the members take part in scheme design; 2 of the members are tasked with preparing and transmitting information; 3 members are responsible for handling issues that arise on WeChat platform. The intervention duration ranges from hospitalization to 6 months postoperatively. In control group, patients are given routine nursing, involving vital signs monitoring, the education of health, the monitoring of postoperative complications, as well as postoperative drainage tube nursing.

### 2.4 Outcomes

The main outcomes is the change of Functional Assessment of Cancer Therapy-Breast version 4.0 (FACT-Bv4.0) score.^[15]^ The evaluation includes social well-being, physical health, functional well-being, and emotional well-being, as well as the “breast cancer-specific additional concerns subscale.” While the secondary result is the numerical rating scale (NRS).

### 2.5 Statistical analysis

Through utilizing the software of IBM SPSS Statistics for Windows, version 20, all the data can be analyzed (IBM Corp., Armonk, NY). Afterwards, all the data are described with appropriate characteristics such as mean, median, standard deviation, as well as percentage. The qualitative parameters for the groups are evaluated by *t* test. The categorical variables are determined by the *χ*^2^ tests. When *P* is less than .05, it is viewed to be significant in statistics.

## 3 Results

Table 1 shows the clinical results between study group and control group.

**Table 1:** Clinical results between study group and control group.

Variables	Study group (n = 50)	Control group (n = 50)	*P*
Physical well-being			
Social well-being			
Emotional well-being			
Functional well-being			
Breast cancer-specific subscale for additional concerns			
Numerical rating scale			
Postoperative complications			

## 4 Discussion

Breast cancer is one of the most prevalent malignancy among female population all over the world.^[16]^ It ranks first in incidence rate and second in mortality. As a result, breast cancer still is a significant issue of public health. The improvement in screening and treatment modalities has made it possible to detect the disease at an early stage. The major treatment for the early breast cancer is surgery accompanied by the adjuvant therapies. Nevertheless, these treatments can lead to adverse reactions containing sleep disturbances, fatigue, and pain. Such effects can markedly worsen a patient's health-related quality of life, particularly during the periods immediately after surgery and adjuvant therapy.^[17]^ Some methods have been utilized for the improvement of the life quality for female breast cancer patients, containing physical activity, the training based on mindfulness, biopsychosocial intervention, as well as group-based intervention.^[18,19]^ Despite these programs have achieved satisfactory results, there is rarely a comprehensive method that involves social, psychological, and physical rehabilitation in postoperative period. With the popularity and wide coverage of mobile Internet access, WeChat, a kind of free communication software based on mobile Internet, has become one of the most widely used social media. Considering that it is not limited via geographical location and time, the multi-mode nursing plan based on WeChat may have significant benefits for patients. Although WeChat can help improve the quality of nursing, the multi-mode nursing project of early rehabilitation of breast cancer patients based on WeChat has not been reported.

## 5 Conclusion

This paper has guided nurses to develop an evidence-based protocol to improve patient care for postoperative women with breast cancer.

## Author contributions

Fang Yin planned the study design. Xiaofang Wu reviewed the study protocol. Yuxia Zhong recruited participants and collected data. Na Zhao wrote the manuscript. All of the authors have read, commented on, and contributed to the submitted manuscript.

**Data curation:** Fang Yin.

**Formal analysis:** Fang Yin.

**Funding acquisition:** Yuxia Zhong.

**Investigation:** Xiaofang Wu.

**Methodology:** Xiaofang Wu.

**Writing – original draft:** Na Zhao, Fang Yin.

**Writing – review & editing:** Fang Yin.
